# Metabolic Consequences of High-Fat Diet Are Attenuated by Suppression of HIF-1α

**DOI:** 10.1371/journal.pone.0046562

**Published:** 2012-10-01

**Authors:** Mi-Kyung Shin, Luciano F. Drager, Qiaoling Yao, Shannon Bevans-Fonti, Doo-Young Yoo, Jonathan C. Jun, Susan Aja, Sanjay Bhanot, Vsevolod Y. Polotsky

**Affiliations:** 1 Division of Pulmonary and Critical Care Medicine, Department of Medicine, Johns Hopkins University School of Medicine, Baltimore, Maryland, United States of America; 2 Department of Neuroscience, Johns Hopkins University School of Medicine, Baltimore, Maryland, United States of America; 3 Hypertension Unit, Heart Institute (InCor), University of Saõ Paulo Medical School, Saõ Paulo, Brazil; 4 Isis Pharmaceuticals, Inc., Carlsbad, California, United States of America; Pennington Biomedical Research Center, United States of America

## Abstract

Obesity is associated with tissue hypoxia and the up-regulation of hypoxia inducible factor 1 alpha (HIF-1α). Prior studies in transgenic mice have shown that HIF-1α plays a role in the metabolic dysfunction associated with obesity. Therefore, we hypothesized that, after the development of diet-induced obesity (DIO), metabolic function could be improved by administration of HIF-1α antisense oligonucleotides (ASO). DIO mice were treated with HIF-1α ASO or with control ASO for 8 weeks and compared with an untreated group. We found that HIF-1α ASO markedly suppressed *Hif-1*α gene expression in adipose tissue and the liver. HIF-1α ASO administration induced weight loss. Final body weight was 41.6±1.4 g in the HIF-1α ASO group *vs* 46.7±0.9 g in the control ASO group and 47.9±0.8 g in untreated mice (p<0.001). HIF-1α ASO increased energy expenditure (13.3±0.6 *vs* 12±0.1 and 11.9±0.4 kcal/kg/hr, respectively, p<0.001) and decreased the respiratory exchange ratio (0.71±0.01 *vs* 0.75±0.01 and 0.76±0.01, respectively, p<0.001), which suggested switching metabolism to fat oxidation. In contrast, HIF-1a ASO had no effect on food intake or activity. HIF-1α ASO treatment decreased fasting blood glucose (195.5±8.4 mg/dl *vs* 239±7.8 mg/dl in the control ASO group and 222±8.2 mg/dl in untreated mice, p<0.01), plasma insulin, hepatic glucose output, and liver fat content. These findings demonstrate that the metabolic consequences of DIO are attenuated by HIF-1α ASO treatment.

## Introduction

HIF-1 is a transcription factor that controls multiple metabolic pathways related to cellular hypoxia [1]–[5]. HIF-1 consists of a constitutively expressed beta subunit and an O_2_ regulated alpha subunit [1], [2]. Exposure to exogenous hypoxia induces insulin resistance, hepatic steatosis, and dyslipidemia [6]–[11], in part through the activation of HIF-1α [12]. Recent studies demonstrated that obesity is associated with adipose tissue hypoxia in humans and rodents [13]–[18]. Adipose tissue hypoxia leads to up-regulation of HIF-1α [16]–[20]. Transgenic mice with constitutive activation of adipose HIF-1α develop mild obesity, insulin resistance and glucose intolerance [19], while mice with tissue-specific knockout of adipose HIF-1α are protected against diet induced obesity (DIO) and metabolic dysfunction [21]. We have recently shown that DIO leads to liver hypoxia [22]. Non-alcoholic and alcohol-induced fatty livers were associated with HIF-1α up-regulation [23]–[26]. Thus, adipose and liver HIF-1α may be an important target to address the metabolic dysfunction of obesity. We hypothesized that the effects of DIO could be ameliorated by pharmacologic HIF-1α inhibition. In this study, we treated DIO mice with HIF-1α anti-sense oligonucleotides (ASO) and examined the impact of this treatment on lipid and glucose metabolism.

## Methods

### Animals

Forty adult male C57BL/6J mice, 6–8 weeks of age were procured from the Jackson Laboratory (Bar Harbor, ME) and housed in a 22°C laboratory with a 12-hr light/dark cycle (light phase 9am–9 pm). Mice were placed on a high fat diet (HFD, TD 03584, Teklad WI, 5.4 kcal/g, 35.2% fat, 58.4% of kcal from fat) for 12 weeks. Thereafter, mice were treated with (1) HIF-1α ASO, 25 mg/kg in 0.2 ml of PBS intraperitoneally (i.p.) twice a week; (2) control ASO, 25 mg/kg in 0.2 ml of PBS i.p. twice a week; (3) or were observed untreated (n = 10 per group), while HFD feeding continued. Body weight and food consumption were measured daily. Upon completion of metabolic measurements and calorimetry, mice were fasted for 5 hrs (8 AM-1PM), bled by cardiac puncture and sacrificed under 1–2% isoflurane anesthesia. Epididymal (EPI), omental (OM), and inguinal (ING) white adipose tissue (WAT) depots, brown adipose tissue (BAT), liver, and skeletal muscle (quadriceps) were harvested. An additional set of mice, also fed HFD for 12 weeks, was utilized for hyperinsulinemic euglycemic clamp testing. These mice were treated with HIF-1α ASO (n = 5) or observed untreated (n = 5) while HFD continued for 8 weeks. The study was approved by the Johns Hopkins University Animal Care and Use Committee (Institutional Animal Care and Use Committee Protocol MO09M351) and complied with the American Physiological Society Guidelines for Animal Studies.

### Anti-sense oligonucleotides (ASO)

ASOs were produced as previously described [27] with modifications. Briefly, rapid throughput screening with 80 ASOs against mouse HIF-1α was performed. The final selection of the HIF-1α ASO (ISIS 298745) was based on the maximal reduction of target gene expression. ISIS 298745 is a 20-mer with the following sequence: GTGCAGTATTGTAGCCAGGC. A chemically identical compound ASO (ISIS 14923) without known complementarity to any known gene sequence was used as a control. Both ASOs were synthesized as 20-base phosphorothioate chimeric ASOs, where bases 1–5 and 16–20 had a 2′-O-(2-methoxyethyl) modification. This chimeric design increased nuclease resistance and mRNA affinity, while maintaining the RNAase H terminating mechanism [27].

### Intraperitoneal Glucose Tolerance Test (IPGTT) and Insulin Tolerance Test (ITT)

IPGTT and ITT were performed during weeks 6 and 7 of ASO treatment in unanesthetized animals. IPGTT was performed after a 5-hour fast by injecting 1 g/kg glucose i.p. Glucose levels were measured by tail-snip technique using a hand-held glucometer (Accu-Check Aviva, Roche, Indianapolis, IN) at baseline and at 10, 20, 30, 60, 90 and 120 minutes after glucose injection. ITT was performed after a 2-hour fast by injecting 0.5 IU/kg insulin (Humulin R, Eli Lilly, Indianapolis, IN, USA) intraperitoneally. Glucose levels were measured as in IPGTT at baseline, and at 10, 20, 30, 40, 50, 60, 90 and 120 minutes post-injection.

*Hyperinsulinemic Euglycemic Clamp* was performed in conscious HIF-1α ASO treated (n = 5) and untreated (n = 5) mice as previously described [28], [29]. Briefly, under 1–2% isoflurane anesthesia catheters (MRE025 Braintree Scientific, Inc., MA) were chronically implanted in the left femoral artery and vein for measurement of blood glucose and infusion of solutions. The catheters were perfused throughout the recovery period by an infusion pump with a sterile saline solution containing heparin (20 U/ml). Animals were allowed 72 h to recover from surgery. Baseline hepatic glucose output was determined by infusing [3-^3^H] glucose (10 µCi bolus + 0.1 µCi/min; NEN Life Science Products Inc.) for 80 min and then acquiring a 100 µl sample to measure [3-^3^H] glucose level. Blood was then centrifuged at 10,000 g and supernatant collected. Red blood cells were resuspended in heparinized saline and reinfused into the mouse. During the subsequent clamp procedure (120 min), [3-^3^H] glucose (0.1 µCi/min) was infused in combination with insulin to assess hepatic glucose output under hyperinsulinemic euglycemic conditions. Whole-body insulin sensitivity was determined by infusing human insulin (20 mU/kg/min; Novalin R, Novo Nordisk, Princeton, NJ) at a constant rate while supplying D50 glucose (Hospira Inc., Lake Forest, IL) at a variable rate through the femoral venous catheter to maintain plasma glucose at 100–125 mg/dl. Blood glucose was sampled from the femoral artery catheter at 10 min intervals with an Accu-Chek Aviva glucometer. The average glucose infusion rate over the last 30 min of the clamp was used to determine insulin sensitivity.

### Metabolic Measurements

Whole-body calorimetry was performed in an open-circuit indirect calorimeter (Oxymax Equal Flow, Columbus Instruments) with 15 chambers. Calorimetry data were collected in four trials (n = 5 mice per group per trial). Injections of HIF-1α ASO and control ASO were continued during this phase of the study, on day 0 at study start, and on day 3. Rates of oxygen consumption (VO_2_, ml/kg/hr) and carbon dioxide production (VCO_2_) were measured for each chamber every 16 minutes throughout the study. Respiratory exchange ratio (RER  =  VCO_2_/VO_2_) was calculated by Oxymax software (v. 4.02) to estimate relative oxidation of carbohydrate (RER = 1.0) versus fat (RER approaching 0.7), not accounting for protein oxidation. Energy expenditure was calculated as EE  =  VO_2_×(3.815+(1.232×RER)) [30], and normalized for subject body mass (kcal/kg/hr).

*Activity Measurements* were performed according to Walston et al. [31]. Briefly, during the last week of the experiment, activity was measured for each mouse daily between 12 pm and 1pm by a trained observer blinded to the treatment group (DYY) [31]. Traveling activity was measured by counting the number of times a mouse completely crossed the cage midline during a 5-minute period. Standing activity was measured by counting each time the mouse balanced itself on its hind paws extending its body vertically onto the wall of the cage or without cage support during a 5-minute period.

### Biochemical Assays

Serum insulin was measured with a Mouse Ultrasensitive ELISA Kit (Alpco, Salem, NH), serum leptin and adiponectin were measured with ELISA kits from R&D Systems (Minneapolis, MN), and liver glycogen and serum glycerol were measured using kits from Biovision (Milpitas, CA). Lipids were extracted from the liver with chloroform-methanol according to Bligh-Dyer procedure. Serum and liver triglyceride and cholesterol, serum free fatty acids and serum β-hydroxybutyrate were measured with kits from Wako Diagnostics, Inc. (Richmond, VA).

*Real Time PCR*: Total RNA was isolated using Trizol Reagent (Life Technologies, Rockville, MD) with additional RNA clean-up using the RNAeasy (Qiagen, Valencia, CA) purification kit. cDNA was produced from total RNA using Advantage RT for PCR kit from Clontech (Palo Alto, CA). cDNA was amplified in real time reverse transcriptase PCR (RT-PCR) with primers from Invitrogen (Carlsbad, CA) and Taqman probes from Applied Biosystems (Foster City, CA). The threshold cycle (Ct) was determined for every sample. The mRNA expression levels were normalized to 18 S rRNA concentrations and quantified according to the 2^−ΔΔCt^ method [32], [33].

### Western Blot

Glycogen synthase kinase (GSK) 3 α and β phosphorylation was measured in liver tissue total lysate by Western blot using GSK-3α, GSK-3β, p-GSK-3α, p-GSK-3β rabbit monoclonal antibodies from Cell Signaling Technology (Danvers, MA) and goat anti-rabbit –HRP conjugate from Biorad (Hercules, CA). HIF-1α protein was measured in nuclear extract of the liver and epididymal white adipose tissue with rabbit polyclonal antibodies from Novus Biologicals (Littleton, CO) and goat anti-rabbit HRP conjugate from Biorad. β-actin was measured with mouse monoclonal antibodies from Abcam (Cambridge, MA), glyceraldehydes-3-phosphate dehydrogenase (GAPDH) was measured with mouse monoclonal antibodies from Sigma (St. Louis, MO) and goat anti-mouse HRP conjugate from Biorad.

### Histology

Liver tissue was fixed in 10% buffered formalin, embedded in paraffin and sectioned into 5 µm slices. Histological slides were stained with periodic acid Schiff (PAS). Histological assessment of tissue morphology was performed using an Olympus microscope (Olympus, Tokyo, Japan).

*Statistical Analyses* were performed using Minitab Statistical Software, release 15 (State College, PA). All values are reported as means ± SEM. For single point measurement, statistical comparisons between three groups of mice were performed using an unpaired *t-*test with Bonferroni correction for multiple comparisons. For multiple measurements, a repeated measure analysis of variance was performed. A p value of less than 0.05 was considered significant.

## Results

### Effect of ASO on HIF-1a transcription

**Figure 1:**
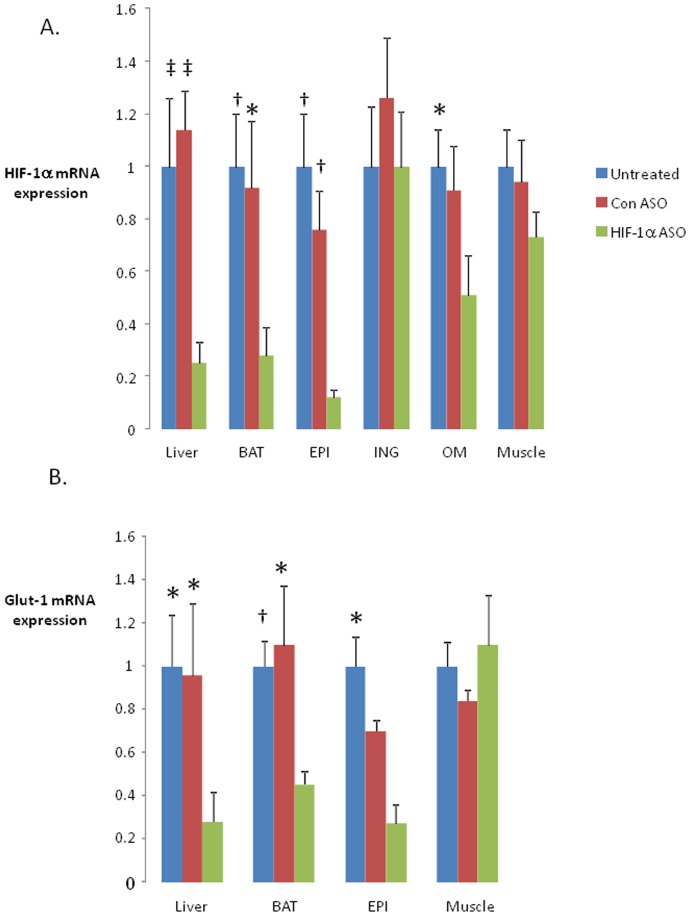
Expression of hypoxia inducible factor 1 alpha (HIF-1α, panel A) and a canonical HIF-1 regulated gene, Glut-1 (panel B) in different tissues of C57BL/6J mice with diet induced obesity (DIO) treated with HIF-1α antisense oligonucleotides (HIF-1α ASO), control ASO or observed untreated for 8 weeks. High fat diet was administered for 12 weeks prior to the ASO treatment and continued during the treatment. BAT, brown adipose tissue; EPI, epididymal adipose tissue; ING, inguinal adipose tissue; OM, omental adipose tissue. *, † and ‡ denote p<0.05, <0.01 and <0.001, respectively, for the difference with HIF-1α ASO treated mice.

**Figure 2:**
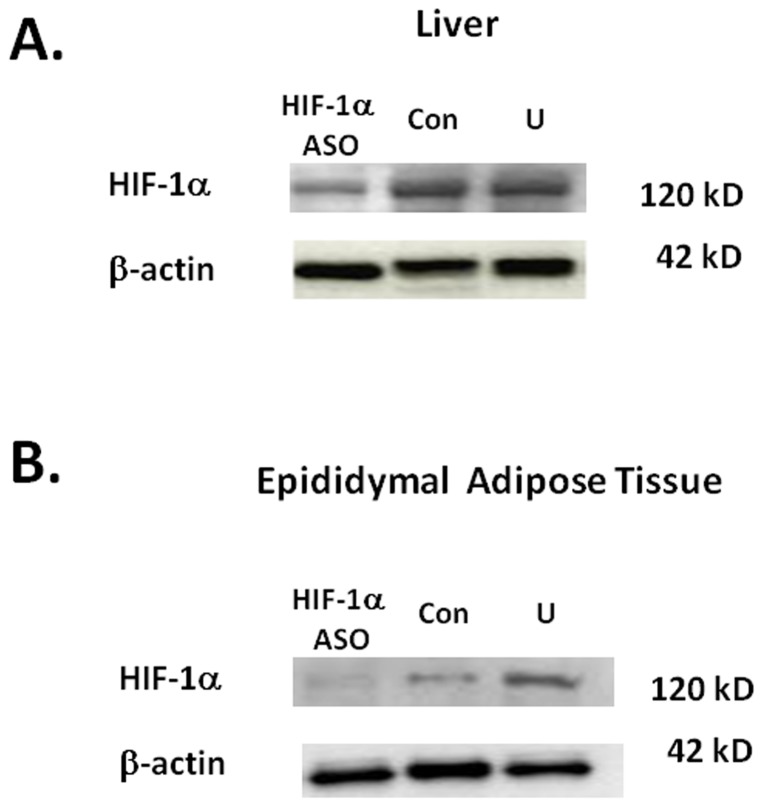
Western blot for HIF-1α in the livers (panel A) and epididymal fat (panel B) of mice with diet-induced obesity (DIO) treated with HIF-1α antisense oligonucleotides (ASO), control ASO (Con) or observed untreated (U) for 8 weeks. N = 5 per group. Representative samples shown. High fat diet was administered for 12 weeks prior to the ASO treatment and continued during the treatment.

HIF-1α ASO decreased HIF-1α mRNA by 75.2±8.3% in the liver, by 72.2±11.2% in brown adipose tissue (BAT), by 88.4±3.4% in epididymal fat (EPI), and by 49±15% in omental fat (OM). HIF-1α expression in inguinal adipose tissue (ING) and skeletal muscle was not altered (Figure 1A). In corresponding tissues, there was a significant decrease in the expression of the HIF-1 regulated gene Glut-1 (1) (Figure 1B). HIF-1α ASO effectively depleted HIF-1α protein from the liver and EPI (Figure 2).

### Effect of ASO on body weight and adiposity

**Figure 3:**
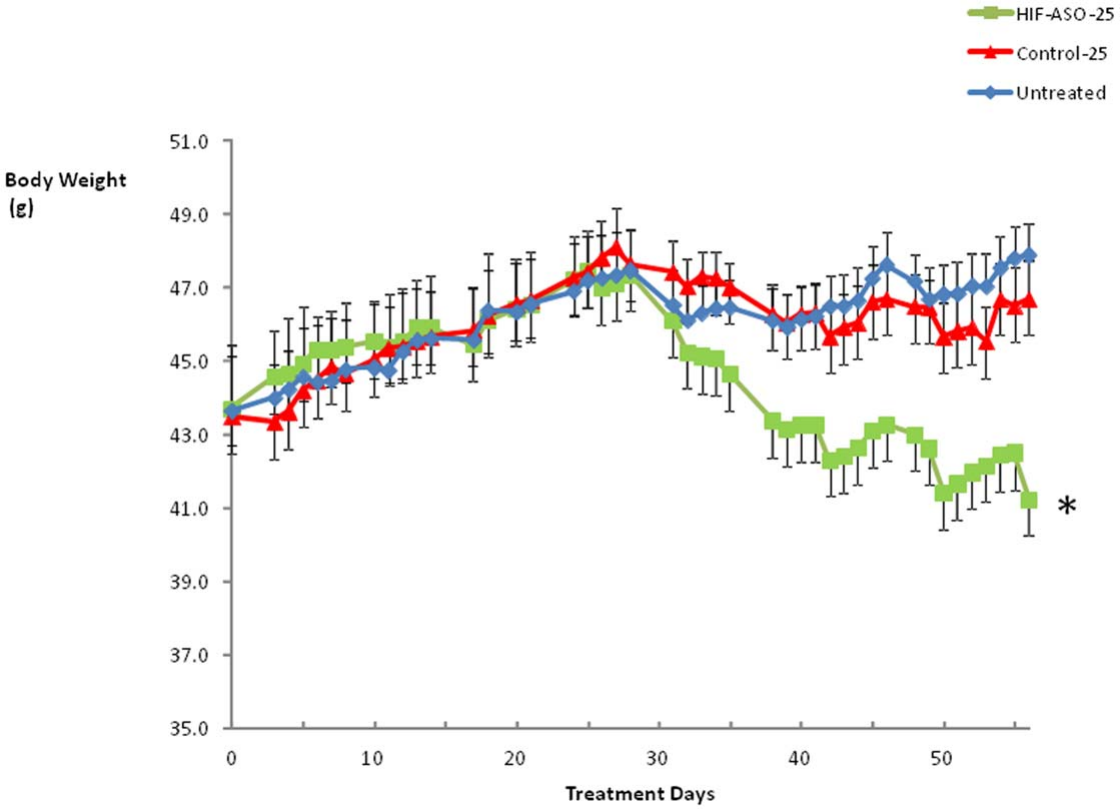
The body weight trajectory of DIO mice treated with HIF-1α ASO, control ASO or observed untreated. * denotes p<0.001 for the difference with HIF-1α ASO treated mice.

HIF-1α ASO treatment led to significant weight loss, observed by the 33rd day of treatment (Figure 3) and the weight curves continued to diverge thereafter. As expected, control ASO treatment did not alter the weight trajectory compared to that of untreated mice. All adipose tissues decreased in weight, including EPI, ING, OM, and BAT (Table 1). Unexpectedly, HIF-1α ASO treated mice exhibited an increase in liver weight.

**Table 1:** Basic Characteristics of DIO mice treated with Hif-1a ASO and control ASO compared to untreated animals.

	Hif-1α ASO treated mice	Control ASO treated mice	Untreated mice
N	10	10	10
Age on Day 0	20.5±2.1 wks	20.5±2.1 wks	20.5±2.1 wks
Body weight on Day 0 (g)	44.6±2.1	43.3±1.6	44.3±1.6
Body weight on Day 56	43.0±1.5	46.5±0.9*	47.2±0.7‡
Average food intake (g/24 hr)	2.7±0.1	2.6±0.1	2.8±0.1
Activity			
Days 49–56: traveling/5 min	12.6±1.4	10.7±1.0	11.4±1.5
standing/5 min	10.2±1.3	11.4±1.3	11.5±1.7
Liver weight (g)	2.13±0.19	1.46±0.10†	1.53±0.11*
Liver/body weight (%)	5.12±0.30	3.17±0.15‡	3.22±0.19‡
Heart weight (g)	0.15±0.004	0.15±0.004	0.16±0.006
Epididymal fat (g)	1.59±0.13	2.05±0.19	2.21±0.19*
Inguinal fat (g)	1.30±0.16	2.31±0.20‡	2.36±0.25†
Omental fat (g)	0.51±0.07	0.70±0.06	0.58±0.04*
Brown fat (g)	0.47±0.05	0.58±0.04	0.66±0.04†
Plasma FFA (mmol/l)	0.32±0.03	0.27±0.11	0.32±0.10
Plasma glycerol (mmol/l)	0.032±0.002	0.036±0.004	0.043±0.008
β-hydroxybutyrate (mmol/l)	0.122±0.014	0.175±0.031	0.150±0.017

* , † and ‡ denote p<0.05, <0.01 and <0.001 respectively for the difference with mice treated with HIF-1α antisense oligonucleotides (ASO).

### Effect of ASO on energy expenditure

**Figure 4:**
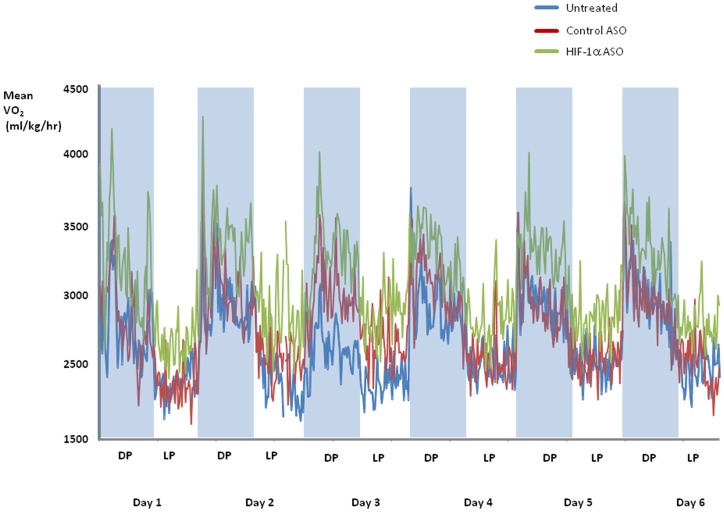
O_2_ consumption (VO_2_) has been analyzed every 16 min for 6 consecutive days in DIO mice treated with HIF-1α ASO, control ASO or observed untreated. Shaded areas represent the 12 hr dark phase (DP) and unshaded areas represent the 12 hr light phase (LP). VO_2_ in HIF-1α ASO treated mice significantly exceeded VO_2_ in mice treated with control ASO and untreated mice, p<0.001. VO_2_ during the dark phase significantly exceeded those during the light phase, p<0.001.

**Figure 5:**
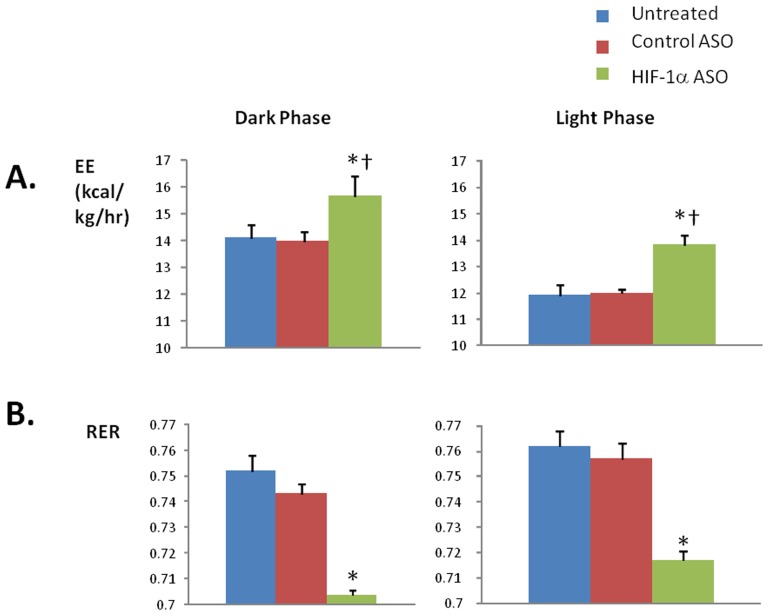
Energy Expenditure (EE, panel A) and Respiratory Exchange Ratio (RER, panel B) in DIO mice treated with HIF-1α ASO, control ASO or observed untreated during the dark and light phases. * denotes p<0.001 for the difference with HIF-1α ASO treated mice; † denotes p<0.001 for the difference with the light phase.

There was no difference in food intake or physical activity between the groups of animals (Table 1). HIF-1α ASO treatment increased VO_2_ and energy expenditure throughout light and dark phases of the day, compared to control ASO and untreated groups (Figures 4 and 5A). ASO treatment lowered the respiratory RER compared to control ASO and untreated groups (Figure 5B).

### Effect of ASO on glucose homestasis

**Figure 6:**
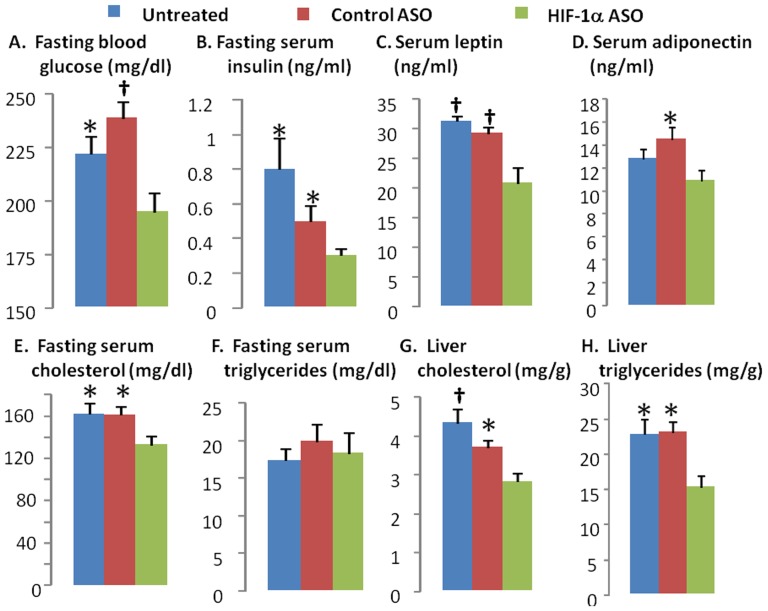
Fasting blood glucose (A) and serum insulin (B), leptin (C), adiponectin (D), fasting serum cholesterol (E) and triglycerides (F), liver cholesterol (G) and triglycerides (H) in DIO mice treated with HIF-1α ASO, control ASO or observed untreated. * and † denote p<0.05 and 0.01, respectively, for the difference with HIF-1α ASO treated mice.

**Figure 7:**
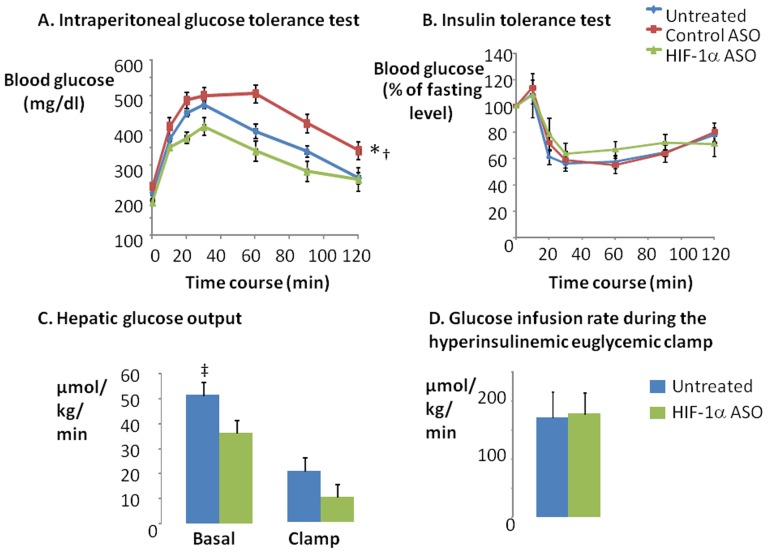
The intraperitoneal glucose tolerance test (A) and insulin tolerance test (B) were performed in DIO mice treated with HIF-1α ASO, control ASO or observed untreated. The hyperinsulinemic euglycemic clamp was performed in HIF-1α ASO treated and untreated mice (C, D). The baseline hepatic glucose output (C) was determined as the ratio of the [^3^H] glucose infusion rate to the specific activity of plasma glucose prior to the clamp. The hepatic glucose output during the clamp was determined as the difference between the ratio of the [^3^H] glucose infusion rate to the specific activity of plasma glucose and non-radioactive glucose infusion rate during last 30 min of the clamp. The whole body insulin sensitivity (D) was measured by glucose infusion rate during last 30 min of hyperinsulinemic euglycemic clamp (glucose levels were clamped at 100–125 mg/dl). * denotes p<0.001 for the difference between untreated or control ASO treated and HIF-1α ASO treated mice; † denotes p<0.001 for the difference between untreated and control ASO treated mice; ‡ denotes p<0.05 for the difference between untreated and HIF-1α ASO treated mice.

HIF-1α ASO treatment significantly decreased fasting blood glucose and insulin levels (Figure 6A, B) suggesting an improvement in insulin resistance. Although there was no difference in glucose tolerance by IPGTT between HIF-1α ASO treated and untreated animals, HIF-1α ASO mice did show improved glucose tolerance compared to the control ASO treated group (Figure 7A). According to the ITT, there was no difference in insulin tolerance between any of the groups (Figure 7B). To clarify discrepancies between fasting values and IPGTT and ITT results, we performed hyperinsulinemic euglycemic clamp in a subset of animals (Figure 7 C, D). HIF-1α ASO treatment significantly decreased hepatic glucose output at baseline conditions (p<0.05). In addition, hepatic glucose output trended lower during the clamp (p = 0.1). In contrast, there was no change in the glucose infusion rate during the clamp. Thus, HIF-1α ASO treatment improved hepatic insulin resistance, without influencing peripheral (skeletal muscle) insulin resistance.

### Effect of ASO on lipid homestasis and liver metabolism

**Figure 8:**
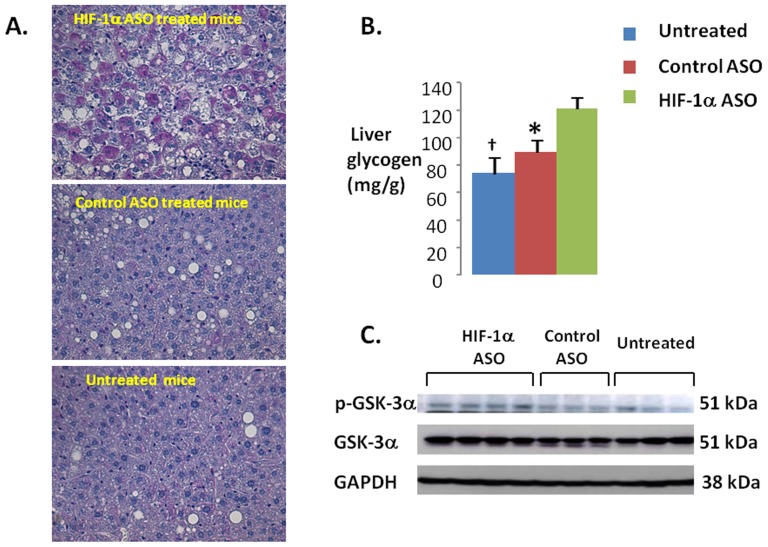
Accumulation of glycogen during HIF-1α ASO treatment. (**A**) Representative images of liver sections in HIF-1α ASO treated (upper panel), control ASO treated (middle panel) and untreated mice (bottom panel). Periodic acid Schiff (PAS) staining, x 400 original magnification. HIF-1α ASO treated mice showed intense PAS positive staining, which was absent in other groups. Control ASO treated and untreated mice show macrovesicular steatosis, which was attenuated in HIF-1α ASO treated mice. (**B**) Glycogen levels in livers measured biochemically (see Methods). * and † denote p<0.05 and <0.01, respectively, for the difference with HIF-1α ASO treated mice. (**C**) Western blots showing phosphorylation of glycogen synthase kinase (GSK) α in HIF-1α ASO treated, control ASO treated and untreated mice in representative samples. GAPDH, glyceraldehydes-3-phosphate dehydrogenase.

**Figure 9:**
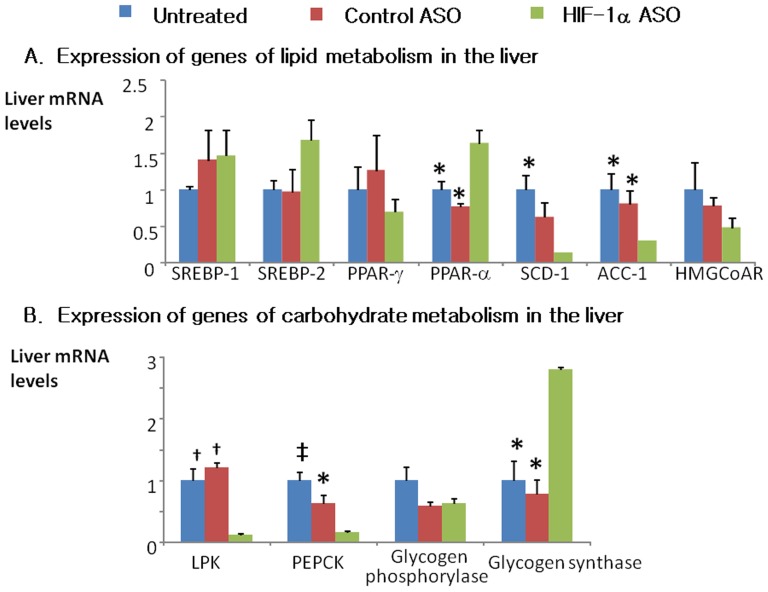
Expression of selected genes of lipid (A) and carbohydrate (B) metabolism in livers of HIF-1α ASO treated, control ASO treated and untreated mice determined by real time reverse transcriptase PCR (RT-PCR). ACC-1, acetyl coenzyme A carboxylase; HMGCoAR, 3-hydroxy-3-methyl-glutaryl-coenzyme A reductase; LPK, liver pyruvate kinase; PEPCK, phosphoenolpyruvate carboxykinase; PPAR, peroxisome proliferator-activated receptor; SCD, stearoyl coenzyme A desaturase; SREBP, sterol regulatory element binding protein. *, † and ‡ denote p<0.05, <0.01 and <0.001, respectively, for the difference with HIF-1α ASO treated mice.

HIF-1α ASO significantly decreased serum leptin, adiponectin, and total cholesterol levels (Figure 6C-E). By contrast, there was no effect of ASO on serum triglycerides (Figure 6F), free fatty acids, glycerol or β-hydroxybutyrate (Table 1). HIF-1α ASO treatment lowered hepatic triglyceride and cholesterol (Fig. 6G, H), while increasing glycogen content, which may account for the observed ASO-induced increase in liver weight (Figure 8A, B). HIF-1α ASO treatment resulted in serine phosphorylation and inactivation of GSK-3 (Figure 8C), which would activate glycogen synthase inducing glycogen biosynthesis [34]. Furthermore, expression of glycogen synthase was increased nearly 3 fold (Fig. 9B).

HIF-1α ASO treatment significantly decreased expression key enzymes of lipid biosynthesis in the liver, including stearoyl co-enzyme A desaturase 1 (SCD-1, 85.6% inhibition) and acetyl coenzyme A carboxylase 1 (ACC-1, 70% inhibition). In contrast, hepatic expression of a key transcription factor of fatty acid oxidation, peroxisome proliferator-activated receptor alpha (PPAR-α) was increased by 64% (Fig. 9A). As expected (1), HIF-1α depletion nearly abolished expression of a key glycolysis enzyme, liver pyruvate kinase (LPK, Fig. 9B). Consistent with a decrease in hepatic glucose output, expression of gluconeogenesis phosphoenolpyruvate carboxykinase (PEPCK) was decreased by 85%. In BAT and WAT, there were no changes in expression of sterol responsive element binding protein 1 (SREBP-1), SCD-1, fatty acid synthase (FAS) or uncoupling protein 1 (UCP-1), whereas BAT UCP-2 was up-regulated (3.19±0.7 compared to 1.25±0.4 and 1.0±0.3 in control ASO-treated and untreated mice, respectively, p<0.05).

## Discussion

The goal of this study was to examine the metabolic impact of HIF-1α inhibition in DIO mice. We showed that chronic intraperitoneal injection of a novel HIF-1α ASO effectively depleted HIF-1α from the liver and adipose tissues. In this setting, we observed several salient metabolic effects of ASO treatment. First, mice exhibited increased energy expenditure and weight loss. Second, hepatic glucose output, fasting glucose and insulin levels decreased. Third, hepatic lipid biosynthetic pathways decreased in association with decreased liver lipids and plasma cholesterol levels. Fourth, HIF-1α ASO increased hepatic glycogen biosynthesis leading to glycogen accumulation in the liver.

These findings are qualitatively similar to previous experiments with transgenic mice, in which HIF-1α knockout in adipose tissue also increased energy expenditure and metabolic uncoupling [21]. The novel finding of our study is that an exogenously administered HIF1-α antagonist can induce similar beneficial metabolic effects, even in the setting of pre-existing DIO. In the discussion, we will review metabolic effects of HIF-1α ASO and elaborate upon implications of our work.

### HIF-1a ASO and Metabolic Rate

HIF-1α ASO caused a significant weight loss starting from Day 33 of treatment. The slow onset of action is characteristic of ASOs, which reduce levels of the target proteins to a new steady state 2–3 weeks after the initiation of treatment and inhibit downstream metabolic pathways 4–6 weeks after the initiation of treatment [35]. For example, DIO mice treated with SCD-1 ASO started losing weight after 4 weeks of therapy [36]. In our present study, HIF-1α ASO-induced weight loss was a result of increased energy expenditure.

HIF-1α ASO increased energy expenditure throughout the day. This phenomenon could reflect increased activity, thermic effects of feeding, or resting metabolic rate. Our data strongly suggest that the increase in metabolic rate was exclusively due to increased resting metabolic rate, since ASO treatment (1) did not affect animal activity or HIF-1α transcription in skeletal muscle; and (2) metabolic rate was uniformly increased during both the dark and light phase, which would not be expected from feeding-induced hyper-metabolism.

Metabolic uncoupling in BAT is one possible cause of decreased metabolic efficiency [37]–[40]. However, HIF-1α ASO did not affect expression of UCP-1 in adipose tissue, which implies that metabolic uncoupling was not implicated [38], [41], [42]. UCP-2 was increased 3 fold, but UCP-2 does not mediate metabolic uncoupling mostly contributing to the control of mitochondrial-derived reactive oxygen species production [43]. Another potential locus of metabolic up-regulation would be the liver. HIF-1 regulates glycolysis (1). ASO effectively depleted HIF-1α from the liver inhibiting Glut 1 transporter and glycolytic enzymes, e.g. liver pyruvate kinase (LPK, Fig. 9B). The inability to utilize glucose switches oxidative processes to burning fat, which indeed occurred as evident from a significant decrease in RER (Fig. 5B). Although we observed up-regulation of a transcription factor of fatty acid oxidation, PPARα (Fig. 9A), activation of fatty acid oxidation could occur at the enzyme activity level to compensate for the lack of glucose utilization. Notably, Jiang examined mice with HIF-1α deficiency in adipocytes (HIF-1α^ΔAdipo^) [21] and found that HIF-1α^ΔAdipo^ mice on a high fat diet had decreased body weight and fat mass compared to wildtype mice, despite similar food intake. HIF-1α^ΔAdipo^ mice exhibited a higher metabolic rate and a decrease in RER [21]. The novelty of our study is that we have shown that therapeutic depletion of HIF-1α in severe obesity induces a metabolic switch to fat oxidation resulting in lower metabolic efficiency, energy wasting and weight loss.

### HIF-1a ASO and Hepatic Lipid Metabolism

We have previously demonstrated that diet-induced hepatic steatosis leads to liver hypoxia, especially in the centers of hepatic lobules (zone 3) [22]. Hepatic steatosis is associated with HIF-1α up-regulation [23]–[26]. We now report that HIF-1α ASO treatment attenuates diet-induced hepatic steatosis. This improvement could be due to the overall decreased adiposity of mice, or a direct stimulating effect of ASO treatment on hepatic lipid oxidation, as mentioned above. In addition, HIF-1α ASO treatment decreased levels of lipid biosynthetic enzymes SCD-1 and ACC-1 in the liver (Fig. 9A). These changes could be caused by decreased insulin resistance and direct transcriptional effects of HIF-1α deficiency [44]–[46]. Of note, despite improvement in hepatic steatosis, HIF-1α ASO increased liver weight (Table 1). The increase in liver weight was likely attributable to accumulation of glycogen, which will be discussed further. Overall, our data suggest that HIF-1α ASO treatment may alleviate hepatic steatosis by increasing lipid oxidation and decreasing *de novo* lipogenesis in the liver.

### HIF-1a ASO and Insulin Resistance

We observed that HIF-1α ASO treatment lowered fasting plasma and insulin levels. Upon further testing of glucose homeostasis with ITT, IPGTT, and the hyperinsulinemic euglycemic clamp, we showed that the ASO treatment lowered hepatic glucose output, but did not alter peripheral insulin action. By contrast, manipulation of HIF-1α in adipose tissue was shown to greatly influence peripheral insulin resistance [19], [21], [47]. Perhaps the incomplete inhibition of HIF-1α in adipose tissue, coupled with suppression of liver HIF-1α, led to the discrepant results in our study. We propose that HIF-1α ASO treatment improved hepatic insulin resistance via two major mechanisms. *First*, HIF-1α depletion attenuated diet-induced hepatic steatosis; hepatic lipotoxicity has been implicated in the development of hepatic insulin resistance [48]–[52], and therefore treatment of lipotoxicity may improve insulin resistance. *Second*, HIF-1α ASO treatment down-regulated a rate-limiting enzyme of gluconeogenesis, PEPCK; transcriptional regulation of liver gluconeogenesis by HIF-1 has been previously reported [53]. Improved hepatic insulin sensitivity results in increased glycogen synthesis. We report an increase in hepatic GSK phosphorylation in HIF-1α ASO treated mice. GSK is one of the major insulin targets in the liver [34]. GSK phosphorylation inactivates the enzyme increasing the activity of glycogen synthase and glycogen synthesis. Transcriptional up-regulation of glycogen synthase may also contribute to hepatic glycogen accumulation in HIF-1α ASO treated mice. In addition, effects of HIF-1α deficiency on glucose metabolism depend on a diet. In mice on a high sucrose diet (40% of the diet composition vs 15% in our study), HIF-1α knockout in the liver increased fasting hyperglycemia and glucose intolerance due to impaired utilization of excessive dietary carbohydrates [26]. Overall, our data suggests that HIF-1α ASO treatment decreases hepatic glucose output and hepatic insulin resistance in DIO.

### Limitations

The purpose of this study was to examine the metabolic impact of a systemic down-regulation of HIF-1α in DIO using novel ASOs. ASO treatment successfully inhibited HIF-1α in multiple tissues, and improved several metabolic pathways in DIO mice. However, the study was unable to identify what tissue or tissues contributed to the observed metabolic phenotype of ASO-treated mice. Another limitation of the study was the lack of significant weight gain in control animals during last 4 weeks of the experiment. This phenomenon can be attributed to a combination of factors, including expected slowing of weight gain in DIO animals after first 12 weeks of HFD [54] and several potentially stressful interventions during the last weeks of the experiment, e.g. IPGTT and ITT. In addition, our study did not show whether HIF-1 regulates genes of lipid metabolism directly, by binding to their promoters, or indirectly, as a result of decreases in plasma insulin and glucose [44]–[46]. Thus, we have shown that HIF-1α ASO treatment causes weight loss, but the mechanisms remain elusive.

### Conclusions and Clinical Implications

We have shown that the depletion of HIF-1α mRNA with ASO (1) leads to weight loss in DIO mice via increased energy expenditure and enhanced oxidation of fat; (2) improves hepatic steatosis, liver insulin resistance and dyslipidemia; (3) induces glycogen accumulation in the liver. HIF-1α ASO could be a candidate for treatment of obesity, fatty liver and metabolic dysfunction in humans.
